# The Role of the ADAMTS18 Gene-Induced Immune Microenvironment in Mouse Kidney Development

**DOI:** 10.3390/biomedicines12020396

**Published:** 2024-02-08

**Authors:** Ben Xu, Jia-En Zhang, Lin Ye, Chang-Wei Yuan

**Affiliations:** Department of Urology, Peking University First Hospital and Institute of Urology, Peking University, Beijing 100034, China

**Keywords:** ADAMTS18, kidney development, immune microenvironment, ureteric bud

## Abstract

The aim of this study is to investigate the role of the ADAMTS18 gene in regulating the renal development of mice. PAS staining was used to observe the kidney development of E12.5–E17.5 mice, while immunofluorescence staining and RT-PCR were used to observe the expression of ADAMTS18. Ureteric bud (UB) branches were observed using immunofluorescence staining using the UB marker E-cadherin, and the apoptosis and proliferation of posterior renal mesenchymal cells were analyzed using TUNEL and PH3 fluorescence staining. Flow cytometry was used to analyze the immune cell infiltration, and western blotting (WB) was used to analyze the expression of PD-1/PD-L1 and CTLA-4. As a result, the ADAMTS18 gene expression gradually increased as the kidney continued to mature during embryonic development. Compared with that in the control and vector groups, UB branching was significantly reduced in the ADAMTS18 deletion group (*p* < 0.05), but that deletion of ADAMTS18 did not affect posterior renal mesenchymal cell proliferation or apoptosis (*p* > 0.05). Compared with those in the control and vector groups, the proportion of embryonic kidney B cells and the proportion of CD8+ cells were significantly greater after ADAMTS18 was knocked down (*p* < 0.05), but the difference in neutrophil counts was not significant (*p* > 0.05). The WB analysis revealed that the PD-1/PD-L1 and CTLA-4 expression was significantly increased after ADAMTS18 was knocked down (*p* < 0.05). In conclusion, the ADAMTS18 gene may be involved in mice kidney development by regulating the immune microenvironment and activating immune checkpoints. Deletion of the ADAMTS18 gene may be unfavorable for kidney development.

## 1. Introduction

Worldwide, an estimated 6% of babies are born each year with severe hereditary defects; of these, approximately 1% have congenital anomalies of the kidney and urinary tract (CAKUT), which include renal anomalies (renal hypoplasia and hydronephrosis) and anomalies of the lower urinary tract (urethral obstruction, bladder and urethral abnormalities, etc.) [1]. CAKUT is a complex genetically heterogeneous developmental disorder with multiple phenotypes, which may be caused by mutations in a single gene that controls early kidney and lower urinary tract development [2]. Abnormalities in kidney development underlie a variety of disorders: kidney formation begins with the ureteric bud (UB), and abnormalities in the branching pattern of the UB may lead to CAKUT. In addition, abnormalities in the branching of the ureter and a reduced number of tubular apices lead to a reduced number of glomeruli, which can lead to an increased risk of hypertension and chronic kidney disease [3]. Therefore, elucidating the molecular mechanisms and gene expression networks that guide kidney development is critical for the clinical management of a wide range of renal diseases.

Mutations in a disintegrin and metalloproteinase with thrombospondin motifs (ADAMTS) family gene have been associated with certain human hereditary disorders, such as the ADAMTS13 mutation that causes hereditary thrombocytopenia-induced purpura [4]. Mutations in ADAMTS2, which encodes a procollagen peptidase, lead to Ehlers–Danlos syndrome, which is characterized by tissue fragility and skin hyperextension [5], while mutations in ADAMTS5, 9, and 20 are involved in limb morphogenesis, cardiovascular development, skin pigmentation, and palatal development [6]. The main causes of this syndrome are tissue fragility and hyperextension of the skin. Recently, it has been proposed that developing mouse kidneys be screened for genes enriched in epithelial branching shortening, including ADAMTS18, and Rutledge et al. reported abnormalities in the reproductive tract, lung, and lens development in mice defective in ADAMTS18 [7]. Other members of the ADAMTS family play important roles in the regulation of a wide range of cellular functions (including extracellular matrix transfer, tissue morphogenesis, blood coagulation, and ovulation) and histopathology (including arthritis, atherosclerosis, cancer, angiogenesis, and wound healing) [8].

The ADAMTS18 gene was first identified and named in 2002 and has been defined as an “orphan ADAMTS protein”—i.e., a member of the ADAMTS family with no known function or substrate. It has been reported in the literature that mice with the deletion of exons 5–6 of the ADAMTS18 gene were crossed with wild-type mice to obtain ADAMTS18 deletion heterozygous mice, and by mating heterozygous mice with each other, ADAMTS18-/- heterozygous mice were obtained; these mice had a deletion of exons 5–6 of the ADAMTS18 gene and were subsequently knocked out by the ADAMTS18 gene [9]. By establishing ADAMTS18 knockout mice, several domestic and international research teams have shown that ADAMTS18 is expressed mainly during early embryonic development and determines the early morphogenesis of many organs through the cleavage and modification of ECM molecules. ADAMTS18 is highly expressed in human embryonic kidneys, which suggests that it may play a role in the development of kidneys. ADAMTS18 has been found to play an important role in the development and morphogenesis of epithelial branching organs such as the lungs, submandibular glands, and lacrimal glands. The kidney is a typical epithelial branching organ, but the exact effect of ADAMTS18 on mammalian kidney development and its underlying mechanism are still unclear. Our group has long been devoted to the study of the ADAMTS18 gene in renal diseases. By establishing an acute renal cell inflammation and necrosis model and a chronic renal fibrosis model in vitro, we proved that the methylation of the ADAMTS18 gene may be related to acute and chronic fibrosis in the kidney. 

The immune microenvironment has a regulatory role in kidney growth and development, and ADAMTS18 was found to be involved in regulating the early branching morphogenesis of the embryonic submandibular salivary glands and to play a role in regulating local inflammation and autoimmune responses and activating myofibroblasts in adult mice [10]. The innate immune system and immune cells are closely related to kidney development and related diseases. Macrophages are immune cells in the immune system that remove foreign, diseased, or damaged cells and promote the growth of the developing kidney [11]. In addition, CD45+ immune cells are involved in promoting cystic kidney disease [12] and cystic nephropathy. However, there is a lack of relevant reports on the correlation between the ADAMTS18-induced immune microenvironment and kidney development in mice.

Therefore, the aim of this study is to investigate the mechanism by which the ADMATS18 gene regulates kidney development in mice through the immune microenvironment, and in turn provide a reference for the clinical treatment of kidney-related diseases.

## 2. Materials and Methods

### 2.1. Laboratory Animals

C57/B6 male mice at 9–10 weeks of age and C57/B6 female mice at 7–8 weeks of age were selected, and the ratio of male to female rats was 3:1 in the same litter. The day on which the vaginal plugs were observed was considered 0.5 days (E0.5d), the pregnant mice were housed in separate cages, and the earliest time of birth was considered 0.5 days (P0.5d). This study was approved by the Animal Ethics Committee of Peking University First Hospital (No. 2023050).

### 2.2. Collection of Embryonic Kidneys

Dissecting microscopy was used to separate E12.5d, E13.5d, E14.5d, E15.5d, E16.5d, and E17.5d embryonic kidneys, which were fixed in 4% paraformaldehyde overnight at 4 °C, washed in PBS, and then transferred to 70% ethanol for white treatment and preparation of paraffin sections.

### 2.3. PAS Staining

Staining was performed according to the instructions of the PAS Staining Kit (Cat. No. 395B-1KT, Sigma Aldrich, St. Louis, MO, USA). The stained slides were mounted using Permount (Cat. No. 17986-01; Electron Microscopy Sciences, Hatfield, PA, USA), observed under a light microscope, and photographed.

The paraffin sections were sequentially immersed in xylene I and II, anhydrous ethanol I and II, 95%, 90%, 80%, and 70% ethanol, soaked in distilled water for 2 min, oxidized with aqueous iodic acid for 10 min, and stained with Schiff’s reagent for 30 min. The sections were restained with hematoxylin for 5 min, dehydrated and permeabilized by using 95% ethanol and anhydrous ethanol, sealed with a neutral tree glue, and observed and photographed under a light microscope.

PAS staining revealed a blue nucleus, while the thylakoid stroma, amyloid material, and fibronectin were purplish, and the number of damaged renal tubules was counted under a light microscope.

### 2.4. Immunofluorescence

Sample tissues were cut, and the well-fixed blocks were placed in 10% formalin for 48 h. The fixed tissues were rinsed under running water to remove residual fixative and impurities. Samples were dehydrated step by step from 50%, 70%, 85%, and 95% to pure alcohol (anhydrous ethanol) for 2 h each. The tissue blocks were placed in an equal volume mixture of pure ethanol and xylene for 2 h and then in pure xylene for 2 h. The blocks of tissue material were macerated in an equal volume mixture of melted paraffin and xylene for 1–2 h and then transferred successively into two melted paraffin solutions for approximately 3 h each; the tissue was embedded and sectioned.

The slides were incubated in an oven at a constant temperature of 65 °C for 30 min, soaked in xylene I for 15 min, soaked in xylene II for 15 min, soaked in 100% alcohol, 95% alcohol, 85% alcohol, or 75% alcohol for 5 min, and rinsed with tap water for 10 min. High-pressure restoration was performed using a buffer solution of 0.01 M sodium citrate for 15 min. After natural cooling, the sections were washed three times with 0.02 M PBS. The primary antibody was added dropwise, and the samples were incubated in a wet box at 4 °C overnight and washed with 0.02 M PBS three times for 3 min each. The appropriate proportion of a diluted fluorescent secondary antibody was added dropwise, after which the samples were incubated in a wet box and left at room temperature for 1 h. The sections were rinsed three times with 0.02 M PBS. 

### 2.5. RT‒PCR

The amplification system was constructed as follows: 10 μL of SYBR Green Mix, 1 μL of upstream primer F, 1 μL of downstream primer R, 11 μL of ddH_2_O, 2 μL of cDNA template, and a total volume of 25 μL. The reaction program was as follows: 95 °C for 10 min (95 °C, 15 s; 55 °C, 45 s) × 40; 95 °C, 15 s; 60 °C, 1 min; 95 °C, 15 s; and 60 °C, 15 s. The reaction program was performed at the same time. The data were analyzed using the instrument’s own software, ABI Prism 7300 SDS software.

### 2.6. TUNEL Staining

MSCs were inoculated into six-well plates, cultured for 24 h, washed three times with PBS, fixed for 30 min with freshly prepared 4% paraformaldehyde, incubated with 0.1% Triton X-100 for 5 min, and stained at 37 °C using a TUNEL kit (Yeasen Biochemical, Shanghai, China). DAPI was used to stain and photograph the nuclei at 37 °C under a microscope (Nikon, Tokyo, Japan).

### 2.7. Phospho-Histone-3 (PH3) Staining

PH3 staining was performed to detect mitotic cells and determine cell proliferation. Rabbit anti-PH3 was diluted with 2% HINGS at 1:400 and stained ON at 4 °C. After washing with PBS, the cells were incubated with a secondary antibody against an Alexa-coupled anti-rabbit antibody, and cell proliferation was visualized under a microscope.

### 2.8. Flow Cytometry

Apoptosis was analyzed via flow cytometry using V-PI membrane-bound protein staining. A total of 1 × 10^5^ cells were inoculated in a 6-well plate and washed with PBS. The cells were digested and resuspended in a binding buffer. The concentration was adjusted to 5 × 10^5^/100 μL, and 5 μL of Annexin V/FITC was added for 10 min in the dark at room temperature. Then, 100 μL of a binding buffer was added, the mixture was mixed, and the cells were stained with 5 μL of PI for 5 min at room temperature. Apoptosis was analyzed via flow cytometry using a FACScan flow cytometer (BD Biosciences, San Diego, CA, USA) within 1 h. The cells were then stained for 5 min at room temperature with 5 μL of PI.

### 2.9. Western Blot

Cells or tissues were lysed using a RIPA buffer, the protein concentration was detected using the BCA method, and 50 μg of protein was separated via 10% SDS‒PAGE electrophoresis. Next, the proteins on the separation gel were transferred to a PVDF membrane using electrotransformation, and the blot was blocked with 5% skim milk. After blocking for 2 h, the proteins were incubated with a primary antibody (rabbit anti-human polyclonal antibody, 1:500) at 4 °C overnight. After equilibration at room temperature, the membrane was washed, and a secondary antibody (goat anti-rabbit, 1:1000) was added. The membrane was incubated in Lightning chemiluminescent reagent for 2 min and then removed and placed in an exposure cassette. The photographic films were exposed to a dark room for 1 min and then developed and fixed. The brightness values of each group of target bands were photographed and analyzed using the LabWorks gel imaging analysis system (170-8270).

### 2.10. Statistical Analysis

In this study, each animal group contained three mice, and the average of the relevant data of the three mice in the same group was taken for a subsequent statistical analysis. SPSS 23.0 was used for the statistical analysis, and GraphPad Prism 8 software (GraphPad Software Company, San Diego, CA, USA) was used to plot graphs. The tests were performed and statistically analyzed using a one-way ANOVA and Duncan’s test, with *p* < 0.05 considered to indicate a statistically significant difference.

## 3. Results

### 3.1. Morphologic Changes in Mouse Kidney Development and ADAMTS18 Expression

**Figure 1:**
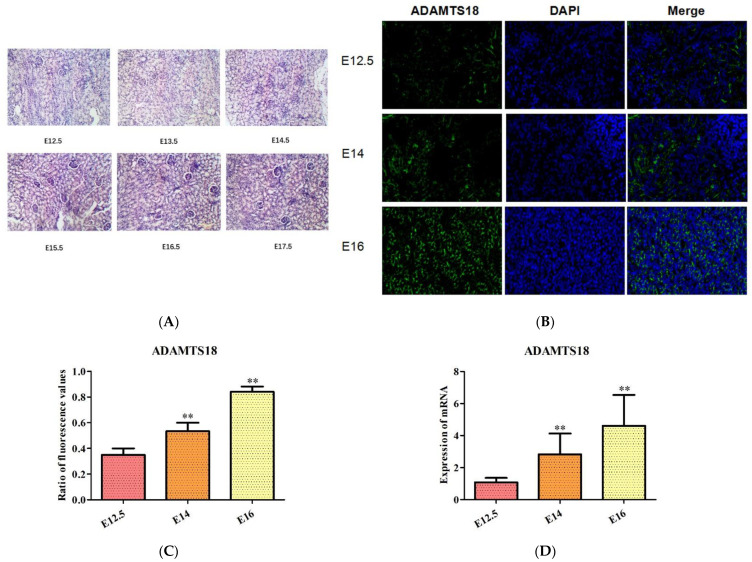
ADAMTS18 expression during mouse embryonic kidney development: (**A**) PAS observation of kidney development in E12.5–E17.5; (**B**) immunofluorescence staining of the posterior renal mesenchyme for ADAMTS18 expression; (**C**) quantitative analysis of the immunofluorescence staining results; (**D**) the RT‒PCR analysis of the relative expression level of the embryonic kidney protein ADAMTS18. ** *p* < 0.05.

Kidney development in E12.5-E17.5 mice was observed via PAS staining (Figure 1A), and the expression level of the ADAMTS18 gene in the posterior renal mesenchyme gradually increased according to immunofluorescence staining as the kidneys continued to mature during the embryonic stage of development (*p* < 0.05) (Figure 1B,C). An RT‒PCR analysis of the expression level of ADAMTS18 at different developmental stages revealed that the kidney expression of ADAMTS18 gradually increased (*p* < 0.05) (Figure 1D).

### 3.2. ADAMTS18 Deletion Induces UB Defects

**Figure 2:**
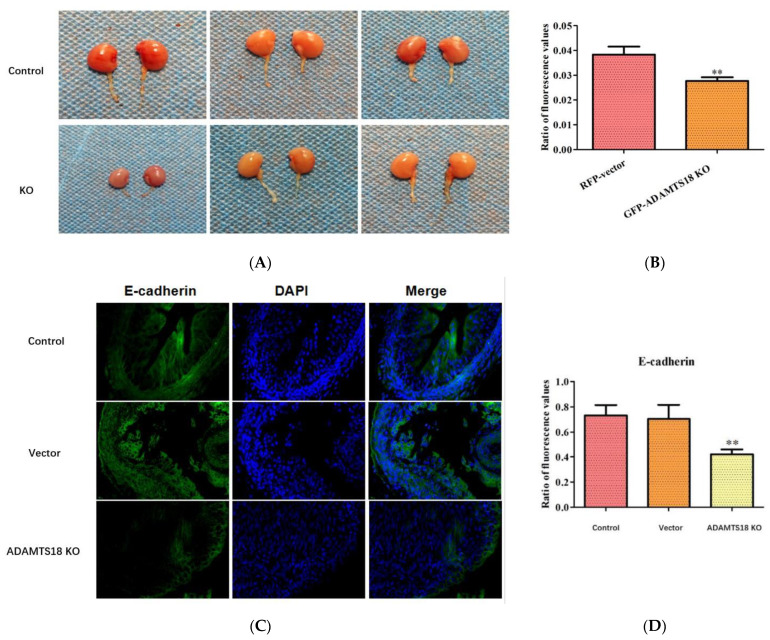
ADAMTS18 deletion induces UB defects (in the GFP-ADAMTS18 KO group and control RFP vector group): (**A**) the appearance of embryonic kidneys and ureter; (**B**) quantitative analysis of the organ weight; (**C**) immunofluorescence staining with E-cadherin to observe UB branching; (**D**) quantitative analysis of UB. ** *p* < 0.05.

Wild-type E14.5d embryonic kidney tissues were microisolated and placed in petri dishes with a DMEM culture medium, and E14.5d embryonic kidneys were cultured in vitro. GFPADAMTS18 KO and control RFP vector cells were microinjected into the posterior renal mesenchyme of embryos. From a weight perspective, it was observed that the kidney development was limited in the GFPADAMTS18 KO group (*p* < 0.05) (Figure 2A,B). UB branching was observed via immunofluorescence staining using the UB marker E-cadherin, which was significantly reduced in the ADAMTS18 deletion group compared with that in the control and vector groups (*p* < 0.05) (Figure 2C,D), suggesting that the knockdown of ADAMTS18 may be detrimental to UB development.

### 3.3. ADAMTS18 Deletion Did Not Affect Postembryonic Renal Mesenchymal Cell (MSCs) Proliferation or Apoptosis

**Figure 3:**
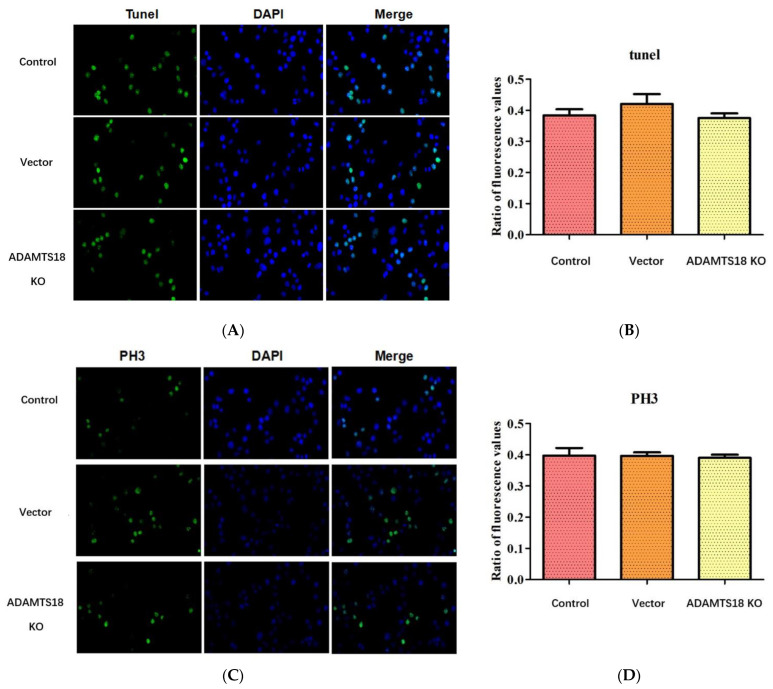
Apoptosis and proliferation of posterior renal MSCs after E14.5 transfection of plasmid cultured in vitro for 3 days: (**A**) TUNEL staining; (**B**) quantitative analysis of TUNEL results; (**C**) PH3 staining; (**D**) quantitative analysis of PH3 results.

Posterior renal MSCs were analyzed for apoptosis and proliferation using TUNEL (Figure 3A,B) and PH3 (Figure 3C,D) fluorescence staining, and the results showed that the deletion of ADAMTS18 did not affect the proliferation or apoptosis of posterior renal MSCs (*p* > 0.05).

### 3.4. Analysis of Immune Cell Infiltration

**Figure 4:**
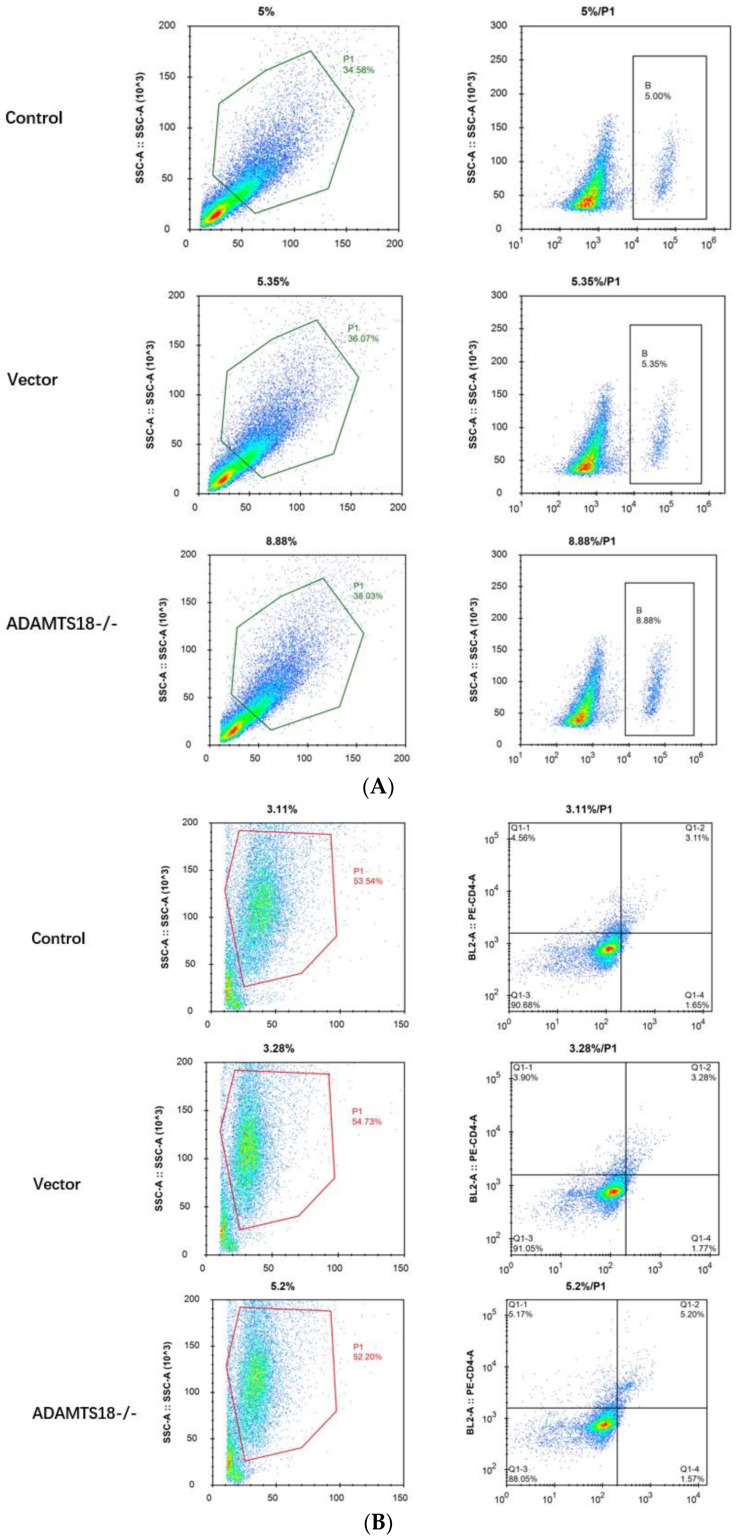

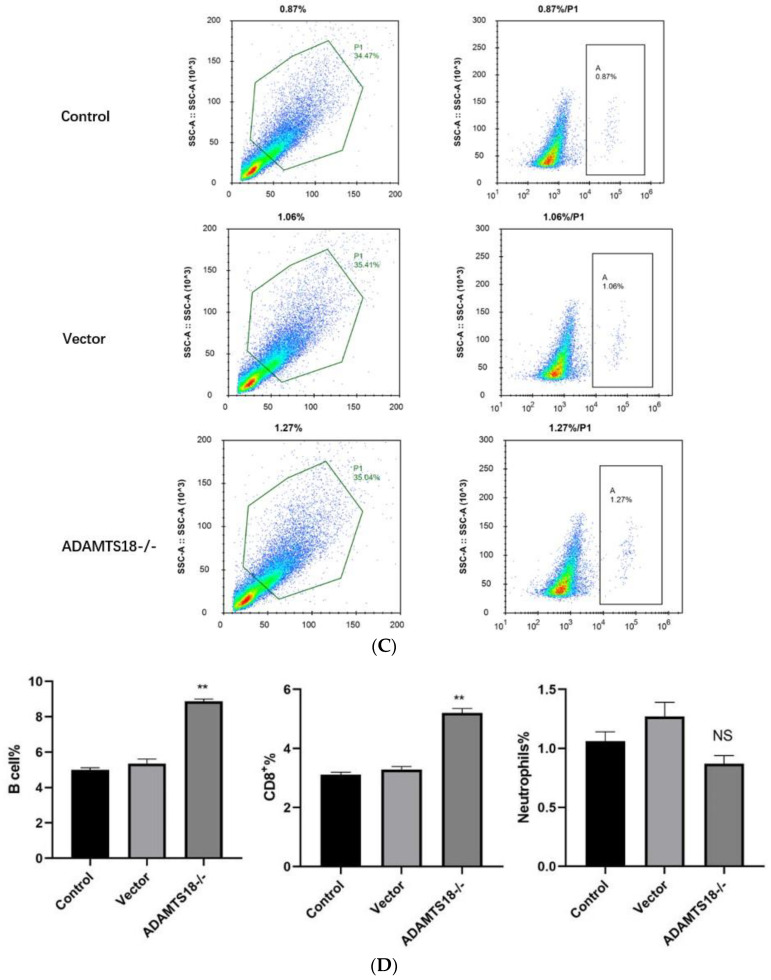
Flow cytometry analysis of embryonic kidney immune cell infiltration: (**A**) B cells; (**B**) CD8 cells; (**C**) neutrophils; (**D**) quantitative analysis of immune cell infiltration results. ** *p* < 0.05. NS = Not Significant.

E14.5d embryonic kidneys were divided into a control group, a vector group, and an ADAMTS18-/- group. A flow cytometry analysis of the B cells, CD8+ T cells, and neutrophils in embryonic kidney tissues was performed to evaluate the effect of ADAMTS18 knockdown on immune cell infiltration. The results showed that, compared with those in the control and vector groups, the proportion of B cells and the proportion of CD8+ cells were significantly greater in the ADAMTS18 knockdown group (*p* < 0.05), but the difference in neutrophil numbers was not significant (*p* > 0.05). The above results suggest that the embryonic kidney ADAMTS18 knockdown may cause immune cell infiltration and immune disorders (Figure 4A–D).

### 3.5. Analysis of ADAMTS18 Expression and Immune Checkpoint Expression

**Figure 5:**
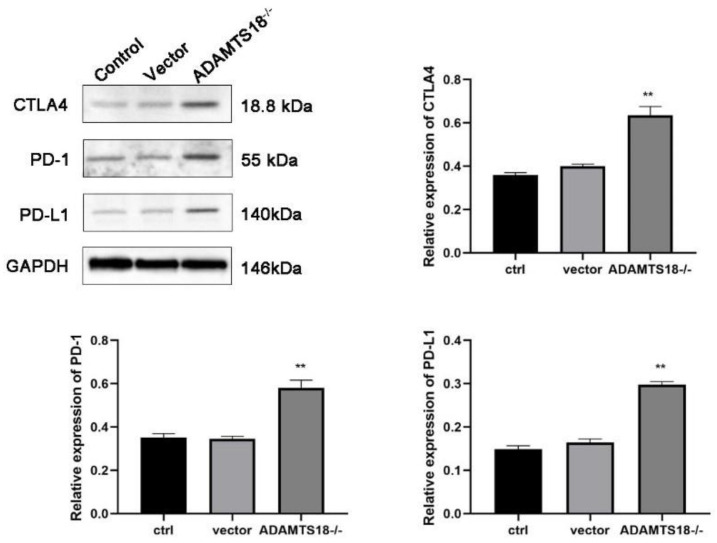
WB analysis of the expression of key immune checkpoint proteins. ** *p* < 0.05.

PD-1/PD-L1 and CTLA-4 are major immune coinhibitory receptors, and immune checkpoints are associated with a variety of diseases, whereas the tissue immune microenvironment influences the normal development of the ureter during ureteral development. Next, we further analyzed the relationship between ADAMTS18 expression and embryonic kidney immune checkpoint expression, and the results showed that at E14.5d, after the embryonic kidney knockdown of ADAMTS18, the PD-1/PD-L1 and CTLA-4 levels were significantly increased, as determined by the WB analysis (*p* < 0.05). The results confirmed that ADAMTS18 knockdown may induce developmental abnormalities by modulating the immune microenvironment and activating immune checkpoints (Figure 5).

## 4. Discussion

The kidney is an important organ in mammals, and the mouse kidney starts to develop at E10.5d. Several cells in the posterior renal mesenchyme release inducible signaling molecules to the Wolffian duct at E10.5d. In addition, under the stimulation of signaling molecules, the caudal end of the Wolffian duct is induced to sprout a small UB [13,14], and the growing UB then starts to extend toward the lax posterior renal mesenchyme and branches during extension. By E11.5d, the UB branches out to form a “T”-like structure [15]. At this point, the initially formed posterior kidney consists of the “T”-shaped UB and the metaneplric mesenchyme (MM), which accumulate around the tip of the UB in the form of a cap (this point is important for the comparison of the MM with the UB). Mutual induction is very important [13], at which point the UB is also established by an unknown mechanism—a molecular mechanism that is capable of maintaining the nephrogenic program in the posterior renal mesenchyme until early postnatal life [16,17,18], when renal development begins to occur in response to reciprocal induction between the UB and MM [19] and ultimately develops and differentiates to form kidneys with intact renal units [20].

Inflammation is an important factor in chronic renal disease, and ADAMTSs are important mediators of inflammation [21,22]. In recent years, several studies have shown that aberrant expression or deletion of several ADAMTS genes is closely associated with renal development and injury; for example, the deletion of ADAMTS1 can lead to neonatal renal insufficiency, calyceal dilatation, or renal fibrillation [23], ADAMTS7 is closely associated with early inflammatory renal injury [24], and ADAMTS13 is associated with renal functional injury [25,26]. Studies have reported that ADAMTS18 is highly expressed in mouse fetal kidneys, suggesting that ADAMTS18 plays some role in renal development and maintenance of renal function and plays an important role in the formation of the glomerular filtration barrier in mice [27]. However, the specific relationship between ADAMTS18, a substrate-unknown orphan protease, and renal development in mice has not been reported.

The results of this study show that with continuous embryonic development, the expression of the ADAMTS18 gene in the kidney increased, and the period of E14.5 was selected for the observation of embryonic kidney development. To analyze the effect of embryonic kidney ADAMTS18 gene defects on the development of kidneys, the kidneys were poorly developed in ADAMTS18-deficient mice although ADAMTS18 deletion did not directly affect postembryonic renal MSCs proliferation or apoptosis, which indicated that some other mechanisms may be involved in this process. Moreover, the knockout of ADAMTS18 mice embryonic kidney B cells and CD8+ cells was significantly greater, while the difference in neutrophil counts was not significant. Additionally, the analysis of immune checkpoints showed that the knockout of ADAMTS18 embryonic kidneys, PD-1/PD-L1 and CTLA-4, was significantly greater, activating immune checkpoints and inducing developmental abnormalities. Generally, 1 week after the birth of a mouse, the UB stops branching, and the glomeruli in the renal units are basically mature. Since the number of renal units (or glomeruli) is determined by the number of UB [28], after this time, the number of renal units no longer increases, and the renal units further mature at this stage. At 2 weeks after birth, the kidney fully develops. Previous studies by our team confirmed the role of the ADAMTS18 gene in renal chronic fibrosis in an in vitro cell line [29] and confirmed the reversal of obstructive fibrosis via overexpression of the ADAMTS18 gene in a rat model with ureteral obstruction [30]. These findings fully indicate that ADAMTS18 is a stage-specific gene closely related to renal development.

The kidneys are tightly regulated by certain genes at different developmental stages, and the abnormal development of the kidneys in this study may be related to the dysregulation of ADAMTS18-targeted transcription. The correct sprouting site of the UB is very important, and sprouting too far from the mouth or tail may result in the failure of the ureter and bladder to connect properly. Abnormal sprouting of UB may be regulated by a key gene. Takahashi et al. suggested that mice with Gdnf-/- or Ret-/- gene deletion fail to sprout or sprout abnormally [31]. Errors in UB branching during renal development generally lead to a series of renal developmental disorders, including a reduced number of renal units and cystic kidney disease. In vitro studies have shown that soluble factors produced by renal mesenchymal differentiation can modify the extracellular matrix and affect the termination of UB branching [32]. In vitro studies have shown that soluble factors produced by renal mesenchymal differentiation can modify the extracellular matrix and influence the termination of UB branching. Therefore, understanding and correcting congenital renal developmental anomalies by examining the mechanism of UB branching morphogenesis and regulation are important. The correct sprouting of the UB requires the precise regulation of multiple factors and genes, and disruption of this balance may lead to ectopic sprouting of the UB and abnormal development of the ureter.

ADAMTS18, an extracellular matrix metalloproteinase, can regulate tissue and organ development and disease by cleaving or modifying the distribution or content of extracellular matrix components and affecting intercellular and cell-matrix interactions [33]. ADAMTS18 and its substrates and regulators are critical for organ morphology and development in the kidneys, lungs, and mammary glands, among others [34,35]. Rutledge et al. suggested that ADAMTS18 is involved in the growth of branched apical progenitor cells in the kidney and that its knockdown may lead to renal hypoplasia [36]. A GO analysis of aberrantly expressed genes in the ureteral epithelium showed that several chemokines, complement components, and metalloproteinase genes are involved in the ureteral immune response or inflammatory progression [3]. It was shown that Esrp plays a role in UB cell branching morphology by regulating CD44 [37]. Therefore, the immune microenvironment plays a role in UB morphology, and a previous study of renal cancer by our research team revealed that the ADAMTS18 gene could play an antitumor role in renal cancer by regulating the infiltration status of immune cells [38]. Podocytes promote glomerular development, counteract intraglomerular pressure, maintain the morphology of vascular collaterals, regulate the glomerular filtration rate, produce VEGF to regulate endothelial cells, participate in inflammatory and immune responses, and synthesize and disassemble glomerular basement membranes. Jeffrey W Pippin et al. reported that transcriptomic and immunohistochemical studies demonstrated that anti-PD-1 antibody treatment improved the healthspan of podocytes [39]. Therefore, it is hypothesized that the immune microenvironment may play an important role in the involvement of podocytes in kidney development. In this study, in ADAMTS18-deficient mice, we found that the immune cell population and CD4+/CD8+ population were significantly increased, and PD-1/PD-L1 and CTLA-4 were significantly increased, which activated immune checkpoints and induced developmental abnormalities. The study of the ADAMTS18 gene was bounded by the year 2015, and most of the studies focused on methylation PCR, expression, sequencing, etc. before 2015. After 2016, gene overexpression and knockdown techniques were used [40], and our team’s focus in this research was to integrate these techniques into a complete presentation.

The present study has several limitations, as it lacked knowledge of the developmental status of the embryonic kidney from the perspective of histological appearance because of the lack of relevant experience and the difficulty relating to the procurement of equipment. More critically, this study used only the adenovirus injection method to construct an ADAMTS18 overexpression model, which was defective. In the future, efforts will be made to conduct experiments with the ADAMTS18 gene overexpression/knockout mouse model to more accurately observe the expression of ADAMTS18 in the development of embryonic kidneys and to explain the molecular mechanism of the role of ADAMTS18 in the development of kidneys. In addition, the present study did not analyze in depth the upstream and downstream molecular mechanisms of ADAMTS18 during kidney development, which is a direction that needs to be improved upon in the future.

## 5. Conclusions

In conclusion, the present study revealed that the ADAMTS18 gene may be involved in mouse kidney development by regulating the immune microenvironment and activating immune checkpoints. Deletion of the ADAMTS18 gene may be detrimental to kidney development.

## Data Availability

The data were available on the request.
